# Acute necrotizing encephalopathy in children with COVID-19: a retrospective study of 12 cases

**DOI:** 10.3389/fneur.2023.1184864

**Published:** 2023-08-02

**Authors:** Xia Lin, Ying Wang, Xiaoying Li, Mohnad Abdalla, Fan Zhang, Chunhua Dong, Weifeng Lu, Xia Liu, Jian Zhang, Kang Ma, Xiang Ma, Qin Jiang

**Affiliations:** ^1^PICU, Children’s Hospital Affiliated to Shandong University, Jinan, China; ^2^PICU, Jinan Children’s Hospital, Jinan, China; ^3^Shandong Provincial Clinical Research Center for Children’s Health and Disease, Jinan, China; ^4^Research Institute of Pediatrics, Children’s Hospital Affiliated to Shandong University, Jinan, China; ^5^Research Institute of Pediatrics, Jinan Children’s Hospital, Jinan, China; ^6^NICU, Children’s Hospital Affiliated to Shandong University, Jinan, China; ^7^NICU, Jinan Children’s Hospital, Jinan, China; ^8^Respiratory Interventional Department, Children’s Hospital Affiliated to Shandong University, Jinan, China; ^9^Respiratory Interventional Department, Jinan Children’s Hospital, Jinan, China; ^10^Department of Respiratory Disease, Children’s Hospital Affiliated to Shandong University, Jinan, China; ^11^Department of Respiratory Disease, Jinan Children’s Hospital, Jinan, China

**Keywords:** acute necrotizing encephalopathy, COVID-19, children, SARS-CoV-2, clinical features

## Abstract

**Background:**

Acute necrotizing encephalopathy (ANE) is a devastating neurologic condition that can arise following a variety of systemic infections, including influenza and SARS-Cov-2. The clinical features of COVID-19-associated ANE in pediatric patients based on multi-case data have not yet been described and remain obscure. We reviewed 12 pediatric patients to better describe the clinical features of ANE with COVID-19.

**Methods:**

We retrospectively collected and summarized the clinical features of ANE in children with COVID-19. Clinical data were collected from 12 children, including their general status, clinical symptoms, laboratory tests, and neuroimaging features.

**Results:**

Among the subjects, 10 were over 5 years old and they accounted for 83.33%. A large percentage of those affected (66.67%) were females. The major manifestations included fever (100%), impaired consciousness (100%), and convulsions (75%). We determined that increased interleukin (IL)-6 and IL-10, and tumor necrosis factor-α and interferon gamma were not predictive of severe ANE and mortality in children with COVID-19 in this study. All children presented with abnormal neuroimaging with multiple and symmetrically distributed lesions, involving the thalamus, basal ganglia, cerebellum, and brain hemispheres. Eight of the 12 children died, resulting in a mortality rate of 66.67%, and 75% of these children were females. Importantly, we found the timely administration of mannitol after an acute onset of convulsions or disturbance of consciousness may be decreased the high mortality induced by ANE children with COVID-19.

**Conclusion:**

COVID-19 associated with ANE in children is characterized by sudden symptom onset, rapid disease progression, and high mortality.

## 1. Introduction

Acute necrotizing encephalopathy (ANE) is characterized by rapid neurological deterioration following a febrile systemic illness; it is most commonly reported in young children. At present, no specific treatments are available for ANE, which has an extremely poor prognosis. Most pediatric patients with ANE are accompanied by different extents of neurological sequelae, and even death. Although the most commonly found infectious trigger is influenza, other pathogens, including severe acute respiratory syndrome coronavirus 2 (SARS-Cov-2), have been associated with ANE (1). COVID-19 is a global public health emergency that has elicited international concern. COVID-19 causes mild to severe illness with high morbidity and mortality. Case reports and data on pediatric COVID-19, particularly those complicated with ANE, have lagged behind those of adults throughout the pandemic. Although several case reports have described ANE complications among children with COVID-19, only a single case was involved (2, 3). The clinical features of COVID-19-associated ANE in pediatric patients based on multi-case data have not been yet described and summarized. Here, we report the clinical manifestations, laboratory tests, magnetic resonance (MR)/computed tomography (CT) imaging and treatment regimen of 12 SARS-CoV-2 infected children with ANE. We also summarize the characteristics to increase awareness and vigilance.

## 2. Methods

### 2.1. General information

From 15 December 2022 to 27 December 2022, a retrospective review was conducted of the clinical, laboratory tests, and MR/CT imaging findings for 12 children admitted to the pediatric intensive care unit (PICU) of the Children’s Hospital Affiliated to Shandong University. The children were determined to be nucleic acid-positive for SARS-CoV-2 by using oropharyngeal swabs.

Sample collection and pathogen identification were performed after admission to the hospital. Nasopharyngeal swabs were collected from all the children and a respiratory pathogen PCR panel including influenza virus, parainfluenza virus, and *Mycoplasma pneumoniae*. The basic information of all the children was recorded, including their age, gender, symptoms, underlying diseases, and vaccination record (Table 1).

**Table 1:** General information of the 12 children with ANE infected by SARS-CoV-2 in Shandong.

No.	Gender	Age	Admit date	Symptom	Underlying disease	Vaccination	Head CT/MR	Prognosis	Time duration from fever to convulsions (day)	Time duration from fever to disturbance of consciousness (day)	Whether to use dehydrating agent immediately after convulsions or disturbance of consciousness
1	F	13 years	2022/12/15	Hyperpyrexia，convulsions，disturbance of consciousness	No	Yes	Positive	Die	2	2	No
2	M	9 years	2022/12/15	Hyperpyrexia，convulsions，disturbance of consciousness	No	Yes	Positive	Die	0.5	0.5	No
3	F	8 years	2022/12/15	Hyperpyrexia，convulsions，disturbance of consciousness	No	Yes	Positive	Coma，turn to the rehabilitation department	1	1	Yes
4	M	1 year 7 months	2022/12/19	Hyperpyrexia，convulsions，disturbance of consciousness	No	No	Positive	Coma，turn to the rehabilitation department	1	1	Yes
5	F	10 years	2022/12/19	Hyperpyrexia，convulsions，disturbance of consciousness	No	Yes	Positive	Die	1	1	No
6	F	11 years	2022/12/21	Hyperpyrexia，convulsions，disturbance of consciousness	No	Yes	Positive	Die	0.5	0.5	No
7	F	13 years	2022/12/22	Hyperpyrexia，disturbance of consciousness	No	Yes	Positive	Die	-	0.5	No
8	M	5 year 5 months	2022/12/23	Hyperpyrexia，disturbance of consciousness，cough	No	Yes	Positive	Die	-	0.5	No
9	F	10 years	2022/12/23	Hyperpyrexia，convulsions，disturbance of consciousness	No	Yes	Positive	coma，turn to the rehabilitation department	1	1	No
10	F	10 years	2022/12/23	Hyperpyrexia，disturbance of consciousness	No	Yes	Positive	Die	-	3	No
11	M	5 years 7 months	2022/12/26	Hyperpyrexia，convulsions，disturbance of consciousness	No	Yes	Positive	come to life，turn to the rehabilitation department	2	2	Yes
12	F	1 year 6 months	2022/12/27	Hyperpyrexia，convulsions，disturbance of consciousness	No	No	Positive	Die	0.5	0.5	No

### 2.2. Laboratory test and imaging examinations

Laboratory test results, including standard blood counts, blood biochemistry, C-reactive protein (CRP), procalcitonin, erythrocyte sedimentation rate, ammo, T cell subsets (CD3^+^, CD4^+^, and CD8^+^), immunoglobulin (IgA, IgM, and IgG), cytokine [interleukin (IL)-2, IL-4, IL-6, IL-10, tumor necrosis factor (TNF)-α, and interferon gamma (IFN-γ)], and myocardial enzyme spectrum, were compiled. Additional data collected included MR/CT imaging, treatment regimens, and prognosis (any severe complications, including death), and recovery condition in progress.

### 2.3. Ethics statement

This study was conducted in accordance with the Declaration of Helsinki. This study was approved by the Ethics Committee of the Children’s Hospital Affiliated to Shandong University. Informed consent was waived because of the retrospective nature of the study and the analysis used anonymous clinical data.

### 2.4. Data analysis

Continuous data were expressed as means and ranges, and categorical data were presented as counts and percentages.

## 3. Results

### 3.1. General information

The pediatric patients included four males (4/12, 33.33%) and eight females (8/12, 66.67%) with a mean age of 8.54 years (ranging from 1.5 to 13 years), as summarized in Table 1. Among them, 10 of the children were over 5 years old (10/12, 83.33%). All the patients were admitted to the PICU of the Children’s Hospital Affiliated to Shandong University and were investigated in this study. All the patients had no underlying disease.

### 3.2. Clinical manifestations

As indicated in Table 1, the primary clinical symptoms of our pediatric patients were fever (12/12, 100%), impaired consciousness (12/12, 100%), convulsions (9/12, 75%), and cough (1/12, 8.33%). The children presented with convulsions and the disturbance of consciousness after having fever for 0.5–2 days and 0.5–3 days, respectively (Table 1).

### 3.3. Laboratory tests and imaging examinations

As presented in Table 2, all the children had normal or decreased white blood cell counts, which is consistent with the major characteristic of viral infection. Eleven children (11/12, 91.67%) had decreased lymphocytes, nine children (9/12, 75%) had decreased thrombocytes, six children (6/12, 50%) had elevated CRP, and 10 children (10/12, 83.33%) had elevated D-dimer. Three children did not undergo the immunoassay for IL examinations. Five children (5/9, 55.56%) exhibited increased IL-6, and seven children (7/9, 77.78%) exhibited increased IL-10. Five children (5/9, 55.56%) had decreased IFN-γ, and the TNF-α level of all the nine children were all within the normal range. The preceding laboratory test results were consistent with the major characteristic of SARS-CoV-2 infection.

**Table 2:** The laboratory results of 12 children.

No.	WBC (10^9^/ L)	N (%)	N (10^9^/ L)	L (%)	L (10^9^/ L)	PLT (10^9^/ L)	CRP (mg/ L)	PCT (ug/L)	D- dimer	APTT (s)	CK-MB (U/L)	CRE (μmol/L)	BUN (mmol/L)	ALT (U/ L)	AST (U/ L)	Amylase (U/L)	BA	CD3 (%)	CD4 (%)	CD8 (%)	IgA (g/L)	IgM (g/L)	IgG (g/L)	IL-2 (pg/ml)	IL-4 (pg/ml)	IL-6 (pg/ml)	IL-10 (pg/ml)	TNF-α (pg/ml)	IFN-γ (pg/ml)	The proportion of abnormal laboratory results
1	4.78	72	3.44	20.90	1.0	183	22.70	1.09	8.7	38.8	6.02	62	4.9	21	53	136	43	47	11.88	28.1	1.87	1.51	8.54	1.08	1.39	36	57.6	1	0.56	0.54
2	26.19	92.70	24.27	3.8	0.99	109	31.61	18.99	107	72.2	51.3	198	15	3,935	6,500	108	70	47	25.61	19.6	1.04	0.64	8.70	2.81	2.94	186.2	148	3	4.49	0.64
3	7.72	91.20	7.06	4.80	0.37	113	7.93	51.56	0.5	39.3	0.8	55	6.7	68	120	63	36	59	35.39	21.4	1.65	0.92	10.70	2.24	2.84	17.91	22.1	3	4.24	0.36
4	10.30	73.60	7.58	23.80	2.45	123	9.35	26.60	11	37.7	5.83	24	7.8	793	881	101	47	67	39.02	25.8	0.36	0.81	7.06	0.3	0.97	5.28	16.5	1	1.76	0.43
5	11.22	84.70	9.51	10.70	1.2	175	5.97	14.64	5.6	36	51.16	54	9.9	394	212	250	26	56	24.09	25.1	1.39	0.0	11.60	0.4	1.06	23.70	16.86	1	1.58	0.57
6	2.36	43.40	1.02	48.10	1.14	123	1.67	0.05	68	180	6.5	76	4.6	8	62	98	71	38	15.63	15.3	1.72	1.98	14.20	-	-	-	-	-	-	0.50
7	12.80	95.80	12.27	2.60	0.33	146	14.92	14.27	8.6	32.30	22.80	51	5.5	888	1,276	56	55	60	36.21	20.3	1.92	1.05	23.70	0.82	1.56	14.71	15	2	1.07	0.46
8	6.03	90.60	5.47	7.50	0.45	86	24.72	66.83	17	60	47.84	66	12	1702	1,437	107	60	50	28.3	17.4	0.81	0.83	7.32	-	-	-	-	-	-	0.64
9	5.33	80.90	4.31	11.60	0.62	120	8.61	3.86	3.2	43.6	3.99	27	3.5	14	42	68	29	46	20.78	22.1	1.01	1.14	8.72	0.74	0.87	2.92	2.09	2	0.56	0.39
10	7.63	93.50	7.14	4.10	0.31	48	94.75	45.24	25	48	94.80	257	17	201	308	126	41	76.9	40.24	35.6	0.89	0.59	22.6	0.56	0.87	65.95	7.22	2	1.3	0.57
11	8.77	91.20	7.99	5.80	0.51	197	3.49	0.19	0.5	35.4	1.52	21	3.5	48	137	67	55	50	22.71	20.2	3.07	1.35	8.87	0.74	0.79	4.19	5.11	1	1.54	0.36
12	2.41	78	1.88	20.30	0.49	57	19.5	132	68	50.9	19.0	104	9.4	4,187	5,976	78	56	37	17.92	17.9	0.40	1.03	5.59	-	-	-	-	-	-	0.73

WBC, White blood cell; N, Neutrophil; L, Lymphocyte; PLT, plate; CRP, C-reactive protein; PCT, procalcitonin; APTT, Activated partial thromboplastin time; CK-MB, creatine kinase MB; CRE, Creatinine; BUN, Blood urea nitrogen; ALT, alanine aminotransferase; AST, aspartate aminotransferase; BA, blood ammonia; IgM, Immunoglobulin M; IgG, Immunoglobulin G; IgA, Immunoglobulin A; IL-2, interleukin 2; IL-4, interleukin 4; IL-6, interleukin 6; IL-10, interleukin 10; TNF-α, tumor necrosis factor α; IFN-γ, interferon-γ; −, not obtained.

In addition, nine children (7/12, 58.33%) showed decreased percentage of CD3^+^ and CD4^+^ T cells. Immunoglobulin levels, including IgA, IgG, and IgM, were all within the normal range. Nine children (9/12, 75%) had elevated CK-MB, creatinine (CRE), and/or blood urea nitrogen (BUN). Eleven children (11/12, 91.67%) showed increased aspartate aminotransferase (AST), while 9 children (9/12, 75%) showed increased alanine aminotransferase (ALT). Blood ammonia was within the normal range in all the cases (12/12, 100%). The chest imaging of all the children indicated no severe infection.

Among the five children who underwent lumbar punctures, three (60%) had cerebrospinal fluid (CSF) with raised protein. Normal cell counts were presented by all the tested pediatric patients. The next-generation sequencing (NGS) from CSF was negative, as indicated in Table 3. CSF antibodies for autoimmune encephalitis were also negative. Real-time polymerase chain reaction (RT-PCR) assay from oropharyngeal swabs were positive for SARS-CoV-2 infection. PCR for other pathogens, such as influenza, parainfluenza viruses and *Mycoplasma pneumoniae*, were all negative in all cases. IgM for herpes simplex virus was negative.

**Table 3:** The CSF and neuroimaging results of 12 children.

No.	CSF protein	CSF WBC (10^9^/L)	CSF Autoimmune encephalitis antibody	CSF NGS	Other pathogens	Neuroimaging results
1	-	-	-	-	Negative	Bilateral thalamus (CT)
2	-	-	-	-	Negative	Bilateral thalamus, intracerebral sac (MRI)
3	1,055	0.001	Negative	Negative	Negative	Bilateral basal ganglia, thalamus, periventricular, brainstem, cerebellum (MRI)
4	273	0.002	Negative	Negative	Negative	Bilateral thalamus, cerebellum (MRI)
5	-	-	-	-	Negative	Bilateral basal ganglia, thalamus, brainstem, brain hemispheres, cerebellum (CT)
6	-	-	-	-	Negative	Bilateral thalamus (CT)
7	-	-	-	-	Negative	Bilateral thalamus, basal ganglia, brainstem, brain hemispheres, cerebellum (MRI)
8	-	-	-	-	Negative	Bilateral thalamus, basal ganglia, brainstem, brain hemispheres, cerebellum (CT)
9	1,144	0.002	Negative	Negative	Negative	Bilateral cerebellum, brainstem, thalamus, basal ganglia (MRI)
10	-	-	-	-	Negative	Diffuse cerebral edema (CT)
11	371	0.003	Negative	Negative	Negative	Bilateral radiating crowns, thalamus, basal ganglia, periventricular, brainstem (MRI)
12	2,635	0.008	Negative	Negative	Negative	Bilateral thalamus, basal ganglia, brainstem, brain hemispheres, cerebellum (MRI)

**Figure 1:**
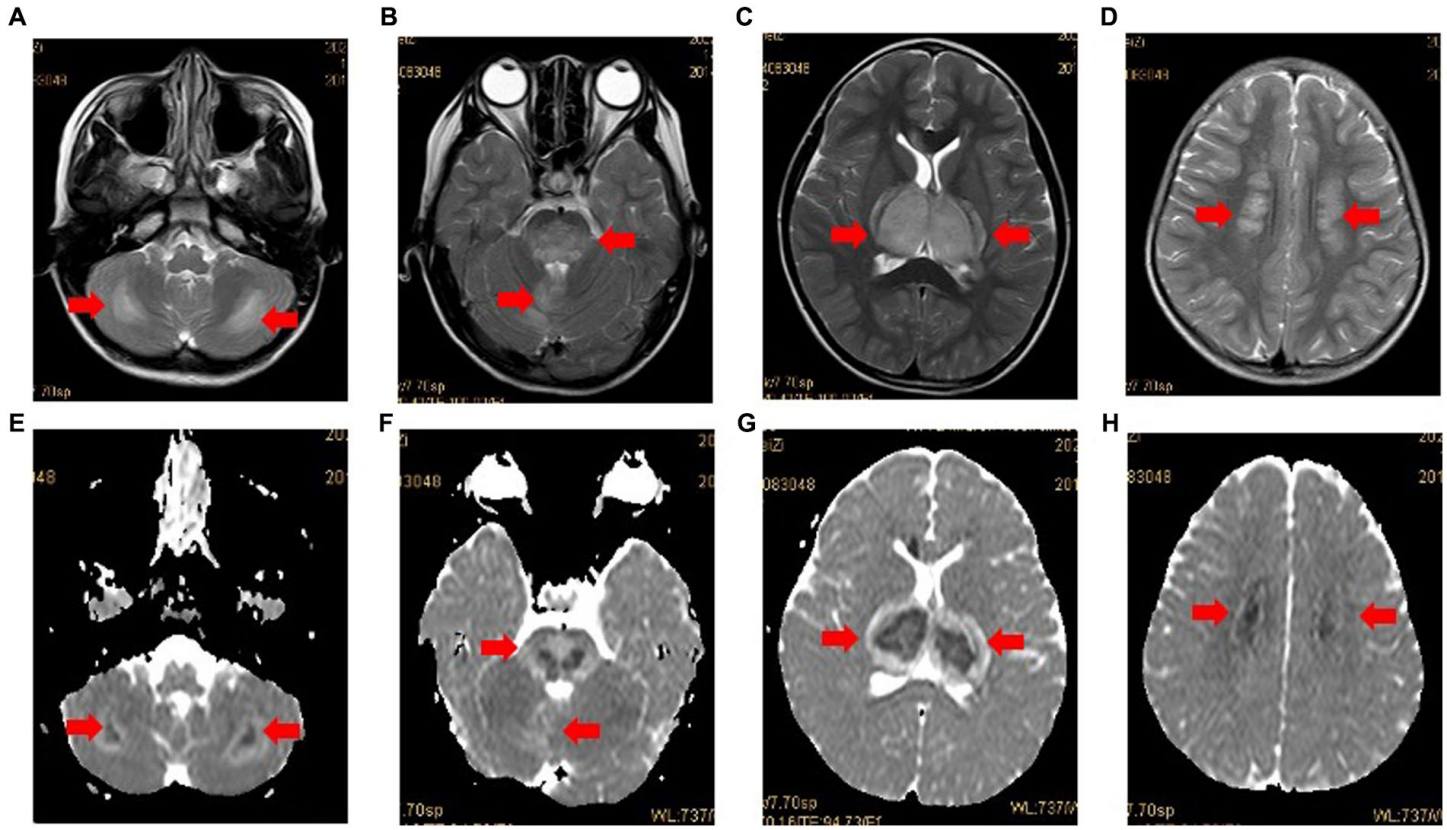
Case 3: MRI in case 3 with typical radiological findings of ANE. Axial T2-weighted images **(A-D)** showing bilateral symmetrical involvement of the cerebellum, brainstem, thalamus and cerebral white matter (arrows), respectively. The apparent diffusion coefficient (ADC) **(E-H)** presenting two gradient modes or there gradient modes.

The diagnosis of ANE was made on the basis of neuroradiological findings according to the criteria proposed by Mizuguchi et al. (4, 5). Eleven pediatric patients (11/12, 91.76%) had multiple and symmetrically distributed lesions (sometimes with hemorrhages) within the thalamus, basal ganglia, and cerebellum on CRI (Figure 1), and one patient (1/12, 8.33%) showed diffuse cerebral edema on CT. These findings were consistent with the primary characteristic of ANE. Four children not only had typical brain lesions (basal ganglia, cerebellum), but also had lesions on their brain hemispheres (Table 3).

### 3.4. Treatment regimen and prognosis

No specific drug is available for treating children with COVID-19. In accordance with the national guidelines on ANE management, all the children required symptomatic sedation (mannitol, glycerin fructose, and 3% sodium chloride) to reduce intracranial pressure after PICU admission. Moreover, the vital signs of the children were monitored, and the children were given symptomatic and supportive treatment. The children were treated with high-dose gamma globulin therapy (1 g/kg/d, total 2 g/kg), high-dose intravenous methylprednisolone (20–30 mg/kg/d, 3 days), and then low-dose intravenous methylprednisolone (1–2 mg/kg, single dose, interval 12 h). Ten children (10/12, 83.33%) required invasive mechanical ventilation.

Eight of the children died. The fatality rate was 66.67%, with 87.5% above 5 years old. Among the eight deaths, 6 (6/8, 75%) of the cases were females and 2 (2/8, 25%) cases were males. The remaining four children (4/12, 33.33%) were transferred from the PICU to the rehabilitation ward. Among them, one child recovered consciousness within 1 week after the onset of ANE and then transferred out of the PICU. Before we submit this article, the three other children were still in coma with convulsive seizures.

All the fatal children were not treated with mannitol immediately after presenting with convulsions or/and the disturbance of consciousness, as shown in Table 1. Three of the four surviving children received prompt mannitol infusion when exhibited signs of convulsions or disturbance of consciousness (Table 1). The administration of mannitol as an antiedematous treatment showed a marked improvement in survival in ANE children caused by Covid-19. In additional, the percent of abnormal laboratory results for the surviving children, including case 3, 4, 9 and 11, ranged 34% from 41% (below 45%), while those of the eight remaining children who died, ranged 45% from 70% (above 45%; Table 2).

## 4. Discussion

ANE is a rare para-infectious syndrome that typically affects children who are suffering from influenza. ANE is characterized by rapidly progressing encephalopathy, seizures, and/or coma caused by multifocal inflammatory central nervous system (CNS) lesions, accompanied by multiple organ dysfunction syndrome and disseminated intravascular coagulation. In recent years, ANE is one of the leading causes of mortality caused by severe influenza in children and it presents severe sequelae.

The global COVID-19 pandemic has resulted in millions of SARS-CoV-2 infections and unprecedented morbidity and mortality (6). Although the respiratory system complications of COVID-19 have been the most frequent and life-threatening, increasing reports indicative of neurological complications (7), including encephalopathy (8), ANE (3), brain microhemorrhages (9), and Guillain–Barré syndrome (10), have emerged.

The direct effect and sequelae of SARS-CoV-2 infection in children and adults lead to devastating neurologic outcomes and significantly high mortality, particularly when the CNS is involved (11). Increasing adult cases of ANE due to SARS-CoV-2 have been previously recognized. However, cases related to pediatric patients have been sporadically reported (2, 3, 12). To the best of our knowledge, this paper is the first report on children with ANE due to COVID-19.

The previous literature suggests that necrotizing encephalopathy is mostly described in cases of influenza virus (13). Peak incidence of influenza-associated encephalitis/encephalopathy is at or below years of 4 years of age, and a large percentage of males are affected (14). However, compared with the influenza virus, our available data showed that COVID-19-associated ANE presented in older children (range: 1.5–13 years), and a large percentage of affected patients were females (8/12, 66.67%). The different age range and sex ratio of children with ANE associated with COVID-19 and other viruses should elicit the attention of physicians.

As expected, 11 pediatric patients (91.76%) exhibited the neuroimaging features of ANE, which are the presence of multiple and symmetrically distributed lesions (sometimes with hemorrhages) within the thalamus, basal ganglia, and cerebellum. One child showed diffuse cerebral edema on CT. In addition, typical brain lesions (basal ganglia and cerebellum) concomitant with the brain hemispheres were observed in four children.

From the five CSF examinations, three pediatric patients (60%) had increased protein level, and all the pediatric patients had normal cell counts. The NGS of the CSF of all the patients presented negative findings. Our findings may suggest that the SARS-CoV-2 virus does not directly infect or attack the brain parenchyma. Thakur et al. conducted RNA scope and immunocytochemistry on brain tissues and did not detect viral RNA and protein in the brain (15). They observed microglial activation, microglial nodules, and neuronophagia in the majority of the brains, but they did not result from direct viral infection of the brain parenchyma, but rather, they were likely from systemic inflammation, perhaps with synergistic contribution from hypoxia/ischemia (15). The mechanism of ANE is extremely complex and not yet clearly known in association with COVID-19, and thus, requires further study.

Increased cytokine levels (IL-6, IL-10, and TNF-α) and decreased IFN-γ expression in CD4^+^ T cells have been associated with severe COVID-19 in adults (16). We found that serum levels of IL-6 (5/9, 55.56%) and IL-10 (7/9, 77.89%) increased. However, IFN-γ (6/9, 66.67%) level decreased in children. This result is not consistent with previous studies. The abnormalities in the cytokine spectrum seen in the current study are consistent with those in a previous study on pediatric patients but not on adult patients with COVID-19. Furthermore, increased IL-6 and IL-10 and decreased IFN-γ were not predictive of severe SARS-CoV-2-associated ANE and mortality in the current study. Children and adults have different inflammatory responses, and thus, additional studies that involve pathogenic mechanisms are necessary. A systemic inflammatory response syndrome that leads to multiple organ failure has been reported in patients with ANE associated with COVID-19. We also found that most of the children had abnormal liver, kidney, and myocardial function tests. This finding is indicative of multiple organ failure. ANE in pediatric patients with COVID-19 accompanied by multiple organ dysfunction, and ultimately, organ failure requires prompt PICU access to monitoring and treatment without delay.

The mechanisms that underlie the neurological complications of COVID-19 remain unclear. The proposed pathogenic mechanisms exhibit the following possibilities: (1) Direct attacks on CNS: Coronaviruses can disrupt the nasal epithelium and cross the epithelial tissue. In certain circumstances that are still not well understood, these viruses reach the bloodstream or lymphatic system, spread to other tissues, and even penetrate the CNS (17, 18). In our study, the CSF of the tested children for virus detection using NGS presented negative findings, which is consistent with the results of most previous studies. It is likely that we have missed the window in which the virus is detectable (19). It is also possible that viral particles in CSF are in undetectable trace amounts (19, 20) and may require a highly sensitive method to test in the future (21); (2) Interaction with angiotensin-converting-enzyme (ACE) receptors: Glial cells and neurons in the CNS have been reported to express ACE2 receptors, making them a potential target of COVID-19 and contributing toward morbidity and mortality. The COVID-19 virus has been found in the general circulation, which may facilitate the virus’ spike protein interacting with ACE2 expressed in the capillary endothelium (22). The subsequent amplification of viral particles’ damage to the endothelial lining leads to propagation toward the brain (22); (3) Cytokine storm: Accumulating evidence suggests that a subgroup of adult patients with severe COVID-19 may have cytokine storm syndrome, which is accounted for the emergence of ANE (23, 24). Experiments on primary glial cultures have shown that they secrete IL-6, IL-12, IL-15, TNF-α, CXCL9, and CXCL10 upon coronavirus infection, indicating that a neurotropic virus infects and activates glial cells and leads to a pro-inflammatory state. The increase in pro-inflammatory cytokines, including IL-6, IFN-γ, and TNF-α, has been proven in patients with COVID-19 and found to be associated with COVID-19 disease severity, and thus, some patients have responded to IL-6 blockade (25, 26). In this study, 5 of the 9 studied children’s IL-6 were mildly increased, and their TNF-α and IFN-γ were within normal range and even decreased in the current research, respectively. Some our findings were consistent with the result of the study of Ran Jia et al. on children with COVID-19 (27). IFN-γ is an important cytokine and plays a vital role in the body’s immunity and defence against viruses (28, 29). It has been shown that this cytokine, when the virus enters the body, inhibits the replication of the virus on the one hand and increases the cytotoxic T lymphocyte killing activity in the body on the other hand (29, 30). Studies in patients have shown that the level of IFN-γ has increased in children with COVID-19, which has not been high compared to adults with COVID-19, this indicates that COVID-19 infection is not severe in children with the disease (29, 31). IFN-γ has potent antiviral activity, and NK cells produce IFN-γ in the early stage of viral infection (3–5 days after infection) to inhibit viral replication (32, 33). However, in the current study the presence of decreased levels of IFN-γ may be indicated that the antiviral activity was impaired in children with ANE at the late stage of SARS-CoV-2 infection. In this study, the increased IL-10 levels suggested that the body attempts to regulate the inflammatory damage caused by other actors in the cytokine storm, which indicates, to a large extent, that the inflammatory state may already be uncontrollable (33, 34). The imbalance between the pro-inflammatory and anti-inflammatory cytokines may be considered one of the major causes of ANE in pediatric patients with COVID-19. (4) Others: Other mechanisms, such as hypoxia, a persistent infection in the CNS, can participate in the induction or exacerbation of ANE outbreaks in children with SARS-CoV-2. Considering the heterogeneity of the clinical presentation of ANE in pediatric patients with COVID-19, one or even several mechanisms are likely involved.

Previous studies have reported that children with COVID-19 are more likely to be asymptomatic or have mild-to-moderate symptoms, with few deaths reported among children globally thus far (35). Notably, our data provide the first direct evidence that ANE in children with COVID-19 is associated with devastating neurologic outcomes and has a higher mortality rate (66.67%), especially among females (75%). Notably, the actual number of deaths due to COVID-19-associated ANE among children may be higher than the previous estimates. Although clinical progress is mild or asymptomatic for the vast majority of children, severe ANE in children with COVID-19 has a high mortality rate and patients are prone to multiple organ failure. In general, the higher the proportion of abnormal indicators was, the greater the possibility was of poor prognosis. Importantly, we found the timely administration of mannitol after an acute onset of convulsions or the disturbance of consciousness may be reversed the high mortality induced by ANE children with COVID-19. Our study, combined with future data, may provide a critical clue for the early recognition and symptomatic treatment of ANE in children with COVID-19 that pediatricians and public health workers cannot overlook.

ANE has no specific treatment, and only symptomatic supportive treatment is typically employed. Most survivors have severe neurological sequelae. Lee et al. conducted a retrospective study and analyzed the clinical data of 10 patients with ANE with a follow-up duration ranging from 1.6 to 5.6 years. The mortality rate was 40%, and only 30% of the patients survived without neurological sequelae (36). By contrast, Lim et al. reported a retrospective review of seven children with ANE in Singapore and found that 75% of their patients with medium risk had no or only mild neurological sequelae (37). We can speculate that the neurological sequelae of children with ANE may progressively lessen or even disappear with time. Therefore, early and timely diagnosis and treatment, particularly rehabilitation treatment, may be the best way to achieve better neurological outcomes.

Our pooled evidence suggests that age range, mortality, gender ratio, and cytokine spectrum are evidently different between pediatric and adult patients, and such differences also exist between ANE from SARS-CoV-2 versus other viruses. Given the high mortality and disability rates of ANE associated with COVID-19, the clinical spectrum and pattern of pediatric patients necessitate further research and more in-depth reports. However, the patients in this report originated from a single center in China, and thus, they might not represent the general COVID-19 population. We hope that our presentation still has reference value for the diagnosis, treatment, and management of children with ANE associated with COVID-19.

Several important limitations of this study should be highlighted. First, the sample size was small. Second, this retrospective study included the children hospitalized in only one center. Further studies with large multi-center samples are needed. During the period that children in rehabilitation, it is necessary to pay attention to the multimodal neuroimaging in post-COVID syndrome and its correlation with cognition. Further research involving a standardized diagnosis and treatment regimen of ANE children with COVID-19 is warranted.

## Data availability statement

The datasets presented in this study can be found in online repositories. The names of the repository/repositories and accession number(s) can be found in the article/supplementary material.

## Ethics statement

The studies involving human participants were reviewed and approved by the Ethics Committee of the Children’s Hospital Affiliated to Shandong University. Written informed consent from the patients/participants or patients/participants' legal guardian/next of kin was not required to participate in this study in accordance with the national legislation and the institutional requirements. Written informed consent was obtained from the individual(s), and minor(s)' legal guardian/next of kin, for the publication of any potentially identifiable images or data included in this article.

## Author contributions

FZ, CD, WL, XLin, JZ, XLiu, and KM: resources, investigation, and data curation. XLin and YW: writing—original draft preparation. YW and MA: writing—review and editing. QJ, XM, and XLi: project administration and supervision. All authors contributed to the article and approved the submitted version.

## Funding

This study was supported by the Science and Technology Project of Jinan Health Commission (2020-4-88).

## Conflict of interest

The authors declare that the research was conducted in the absence of any commercial or financial relationships that could be construed as a potential conflict of interest.

## Publisher’s note

All claims expressed in this article are solely those of the authors and do not necessarily represent those of their affiliated organizations, or those of the publisher, the editors and the reviewers. Any product that may be evaluated in this article, or claim that may be made by its manufacturer, is not guaranteed or endorsed by the publisher.
